# Environmental Adaptability and Energy Investment Strategy of Different *Cunninghamia lanceolata* Clones Based on Leaf Calorific Value and Construction Cost Characteristics

**DOI:** 10.3390/plants12142723

**Published:** 2023-07-21

**Authors:** Nana Li, Yue Cao, Jinghui Wu, Ting Zhang, Xianhua Zou, Xiangqing Ma, Pengfei Wu

**Affiliations:** 1 College of Forestry, Fujian Agriculture and Forestry University, Fuzhou 350002, China; fafulinana@126.com (N.L.); cyue2020@126.com (Y.C.); zhangting8519@163.com (T.Z.); lxymxq@126.com (X.M.); 2 Chinese Fir Engineering Technology Research Center of the State Forestry and Grassland Administration, Fuzhou 350002, China; zhouxianhua111@163.com; 3 Fujian Shanghang Baisha Forestry Farm, Longyan 364205, China; 27wjh@163.com

**Keywords:** calorific value, Chinese fir, climate change, leaf functional traits, subtropical region, utilization strategy

## Abstract

The calorific value and construction cost of leaves reflect the utilization strategy of plants for environmental resources. Their genetic characteristics and leaf functional traits as well as climate change affect the calorific values. This study explores the differences in energy investment strategies and the response characteristics of energy utilization in leaves to climate change among nine clones of Chinese fir (*Cunninghamia lanceolata*). Considering the objectives, the differences in the energy utilization strategies were analyzed by determining the leaf nutrients, specific leaf area, and leaf calorific value and by calculating the construction cost. The results showed a significant difference in the ash-free calorific value and construction cost of leaves among different Chinese fir clones (*p* < 0.05). There were also significant differences in leaf carbon (C) content, leaf nitrogen (N) content, specific leaf area, and ash content. The correlation analysis showed that leaves’ ash-free calorific value and construction cost were positively correlated with the C content. Principal component analysis (PCA) showed that P2 is inclined to the “fast investment return” energy investment strategy, while L27 is inclined to the “slow investment return” energy investment strategy. Redundancy analysis (RDA) indicates that the monthly average temperature strongly correlates positively with leaf construction cost, N content, and specific leaf area. The monthly average precipitation positively impacts the ash-free calorific value and construction cost of leaves. In conclusion, there are obvious differences in energy investment strategies among different Chinese fir clones. When temperature and precipitation change, Chinese fir leaves can adjust their energy investment to adapt to environmental changes. In the future, attention should be paid to the impact of climate change–related aspects on the growth and development of Chinese fir plantations.

## 1. Introduction

Chinese fir (*Cunninghamia lanceolata* (Lamb.) Hook) is one of the crucial fast-growing afforestation tree species in China, having high ecological and economic value, and plays a vital role in forestry production in China [1]. However, due to global warming, the frequency of seasonal drought in the subtropical region is increasing, and uneven precipitation leads to poor growth or disease in young Chinese fir plantations [2], significantly affecting productivity [3]. Therefore, studying the adaptive characteristics of Chinese fir under climate change is imperative. Previous studies showed that the calorific value and construction cost of leaves could reflect the utilization efficiency of plants for resources and the environment. The energy utilization strategy of plants could also be pivotal, combined with the other physiological indicators, to assess the adaptability of plants to the environment [4]. The calorific value reflected the light utilization efficiency of plants and became an important indicator for measuring their physiological activities [5]. Studies have found that the composition, structure, and function of different plants as well as climate change influence differences in plant calorific values. Changes such as prolonged sunlight, high light radiation, and precipitation can affect photosynthesis and energy fixation processes [6].

The leaf construction cost is necessary for plants to obtain carbon (C) [7]. The change in leaf construction cost among different plant species indicates the energy utilization strategy and the adaptability of plants to the environment [8]. The lower leaf construction cost of plants means that their energy consumption in building photosynthetic areas was relatively small, indicating that plants have higher energy utilization efficiency and growth rate [9], and the higher leaf construction cost is directly related to the stress response of plants [10]. The leaf construction cost is mainly affected by environmental factors such as light intensity and water content. Key functional characteristics of leaves, such as specific leaf area, C content, and nitrogen (N) content, also affect the cost of leaf construction [11].

To date, most studies have focused on analyzing Chinese fir leaves’ morphological and physiological characteristics [12], while research on energy investment strategies and their response to climate change based on calorific value and construction cost is relatively scarce. Therefore, we selected one-year-old Chinese fir stands of nine different clones (L27, S4, S18, S22, P2, P17, P302, Yang020, and Yang061). Through earlier experiments conducted by our research group, significant differences were observed in the structural characteristics of the leaves of these nine clones, and they exhibited a high-temperature and drought response mechanism that resisted strong light and reduced water dissipation.

To further explore the response mechanism of nine different clones of Chinese fir to climate change from the perspective of energy investment, this study measured the leaf calorific value, ash content, C content, N content, and specific leaf area of different clones, calculated their construction costs, and analyzed the impact of climate change on the energy investment strategy and environmental adaptability of Chinese fir leaves. The following hypothesis was proposed; significant differences in the calorific value and leaf construction cost of different clones were related to the C and N content. A significant positive correlation exists between specific leaf areas and other related indicators. Changes in temperature and precipitation can affect the energy investment of Chinese fir leaves.

## 2. Results

### 2.1. Ash-Free Caloric Value and Construction Cost of Leaves among Different Chinese Fir Clones

**Figure 1:**
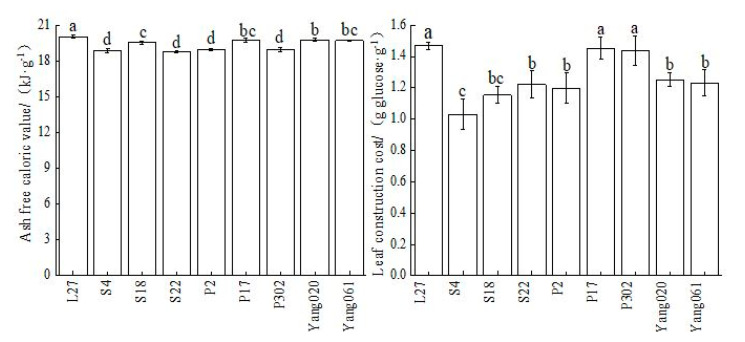
Comparison of leaves’ ash-free calorific value (AFCV) and leaf construction cost (CC_m_) among Chinese fir clones. Different lowercase letters indicate significant differences among clones (*p* < 0.05).

The Chinese fir leaves’ average ash-free caloric value was 19.37 KJ/g, and the variation range was 18.85–20.02 KJ/g. The ash-free caloric value of each clone reached a significant level, among which L27, P17, Yang020, and Yang061 were significantly higher, and S22 was the lowest. The size order of different clones was L27 > Yang 020 > Yang061 > P17 > S18 > P302 > P2 > S4 > S22 (Figure 1). The average leaf construction cost was 1.27 g glucose·g^−1^ among all the clones. The size order of the different clones was L27 > P17 > P302 > Yang 020 > Yang 061 > S22 > P2 > S18 > S4 (Figure 1).

### 2.2. C Content, N Content, Specific Leaf Area, and Ash Content of Leaves

**Figure 2:**
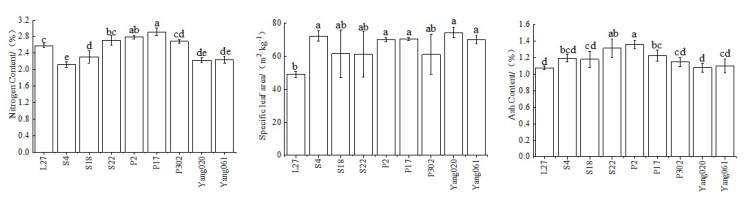
Comparison of nitrogen content (N_mass_), specific leaf area (SLA), and ash content (AC) of leaves among different Chinese fir clones. Different lowercase letters indicate significant differences among clones (*p* < 0.05).

Significant differences were observed in the C content, N content, specific leaf area, and ash content of the leaves among different Chinese fir clones. The leaf C content of clone L27 was higher, and S4 was the lowest (*p* < 0.05) (Table 1). The leaf N content of all the clones ranged from 2.12% to 2.91%, with P17 being the highest and S4 being the lowest. The specific leaf area of Yang020 was the highest, and L27 was the lowest, with a difference of 25.2 m^2^·kg^−1^. The ash content was highest in clone P2 (Figure 2).

### 2.3. Correlation between Ash-Free Caloric Value, Construction Cost, and Other Indexes

**Figure 3:**
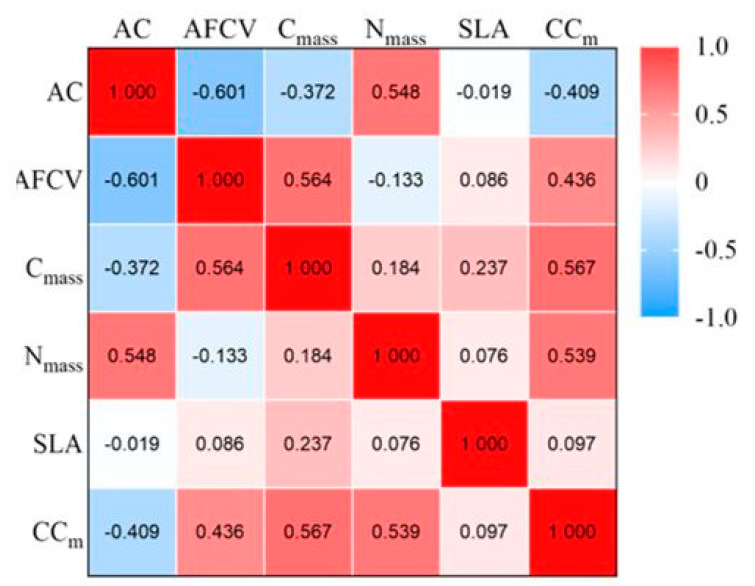
Correlation heat map between different indexes. The numbers in the figure represent the correlation coefficients between different indicators, and the darker the color, the stronger the correlation. AC is ash content, AFCV is ash-free calorific value, C_mass_ is carbon content, N_mass_ is nitrogen content, SLA is specific leaf area, and CC_m_ is leaf construction cost.

Figure 3 shows that AFCV was positively correlated with C content but negatively related to AC (*p* < 0.05). There was no significant correlation between N content and SLA. The construction cost of leaves was positively correlated with ash-free caloric value and C and N content and negatively associated with ash content (*p* < 0.05).

### 2.4. PCA of Indexes of Different Clones of Chinese Fir

**Figure 4:**
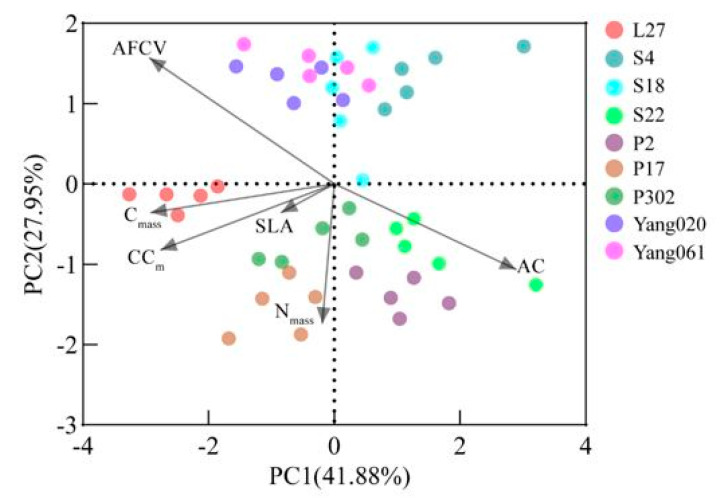
Principal component analysis (PCA) of leaf indexes of different clones of Chinese fir. AC is ash content, AFCV is ash-free calorific value, C_mass_ is carbon content, N_mass_ is nitrogen content, SLA is specific leaf area, and CC_m_ is leaf construction cost.

PCA analysis showed that the variance interpretation rate of PC1 and PC2 accounted for 41.88% and 27.95% of the total variance, respectively (Figure 4), and the cumulative contribution rate was 69.83%. The indicators highly related to PC1 include AFCV, CC_m_, C_mass_, and AC. N_mass_ is highly related to PC2 and negatively correlated with the principal component axis. According to the scores of different clones on the two principal components, L27 has the highest score on the negative half-axis of PC1, and P2 and P17 have the highest score on the negative half-axis of PC2; that is, L27 has a high ash-free calorific value, construction cost, and C content of leaves, while P2 and P17 have high leaf N content. The loading matrix and scores of each indicator are shown in Table 2 and Table 3.

### 2.5. Correlation Analysis between Chinese Fir Leaf Indexes and Environmental Factors

**Figure 5:**
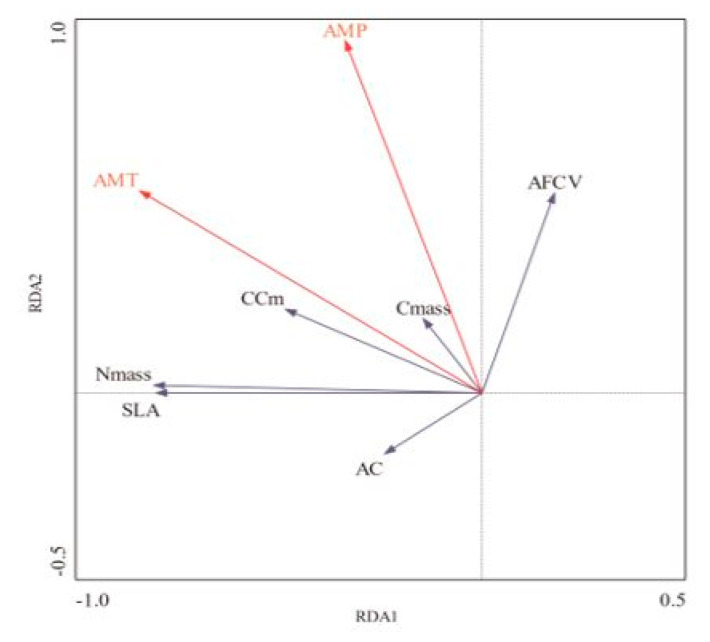
Double sequence diagram of Redundancy analysis (RDA) between leaf parameters and environmental factors. The arrow direction of the hollow vector represents the positive and negative relationship with the sort axis. The longer the vector line with the smaller angle, the more dominant the factor. AC is ash content, AFCV is ash-free calorific value, C_mass_ is carbon content, N_mass_ is nitrogen content, SLA is specific leaf area, CC_m_ is leaf construction cost, AMT is annual mean temperature, and AMP is annual mean precipitation.

RDA can be used to explore the response relationship between leaf index changes and climate factors. As shown in Figure 5, red arrows represent climate factors, and the cosine projection of monthly average temperature on leaf construction cost, N content, and specific leaf area is relatively long, indicating that monthly average temperature has a more significant positive impact on leaf construction cost, N content, and specific leaf area. The monthly average precipitation has a strong positive relationship with the ash-free calorific value and construction cost of leaves. With increase in precipitation, the ash-free calorific value and construction cost of leaves increase, and more energy is invested in the construction of leaves to increase their construction cost.

## 3. Discussion

### 3.1. Energy Investment Strategies for Different Clones of Chinese Fir Based on Calorific Value and Construction Cost

Leaf calorific value can reflect the transformation efficiency of plants to light energy [13]. In this study, the average ash-free calorific value of Chinese fir leaves was 19.37 KJ/g, higher than that of broadleaf forests in subtropical areas [5]. The possible reason for this is that the oil content, lignin, and other high-energy substances in needle trees are high, resulting in a high calorific value [14]. The ash-free calorific value of the leaves of different clones differed significantly; values of L27 and Yang020 were significantly higher than other clones, indicating that the energy utilization strategies of L27 and Yang 020 tended to stabilize energy storage and adapt to the growth environment with more synthesized energetic substances, with strong environmental adaptability. When plants are under environmental stress, the construction cost significantly increases, which is beneficial for plants to resist adverse environments and prolong their leaf life.

High construction cost represents high-stress resistance [15], while low construction cost is closely related to the growth rate of plants [16]. The difference in leaf construction cost among different Chinese fir clones was obvious, indicating differences in energy utilization strategies among different Chinese fir clones [17]. The construction cost of S4, S18, S22, P2, Yang020, and Yang061 was significantly lower than that of L27, P17, and P302, indicating that the former proliferates to obtain more resources. At the same time, the latter has strong resilience and adaptability to adverse environments.

This study shows a significant correlation between the indexes of Chinese fir leaves. The ash-free calorific value and construction cost were positively correlated with C content but negatively correlated with ash content. High C content means increased C-based substances such as lignin, starch, and other compounds [18]; the energy required for synthesizing such substances was high, thus showing higher calorific value and construction cost [19]. Clone L27 has the highest leaf C content, indicating that it has higher energy investment and higher content of high-energy substances such as lipids and proteins than other clones. The ash accumulation does not require a direct energy supply, and increasing its content can reduce plants’ material and energy consumption by building themselves [20]. The ash content can also reflect the accumulation of mineral elements in plants. The ash content of P2 is significantly higher than that of other clones, indicating that it has lower energy investment and a strong ability to enrich mineral elements.

The N content of P17 is higher than that of other clones, and its construction cost of leaves is also higher. High leaf N content corresponds to the “rapid investment return” strategy [21,22,23]. Moreover, the construction cost of leaves is positively correlated with the N content. It has been found that N in leaves is mainly used for protein construction, and the increase in leaf protein content reduces the overall ratio of unstructured C and N, increasing the proportion of high-energy investment components and thus increasing the construction cost of leaves [24,25].

The ash-free calorific value, construction cost, and nutrient content of different tree species could comprehensively reflect the differentiation of their growth strategies [26]. The construction cost can theoretically represent the balance of various factors affecting plant growth. Lower construction cost indicates that plants need less energy to build the same photosynthetic area, and the plants behave in a “fast investment return” strategy. High construction cost positively correlates with the leaf life and the plant shows a “slow investment income” strategy [27]. In the case of limited energy, reduced energy consumption can be used for breeding and other behaviors conducive to species diffusion [28,29].

The PCA showed that different clones’ energy investment strategies differed. L27 is mainly distributed at one end of the PC1 shaft, with high ash-free calorific value, construction cost, and C content, indicating that L27’s energy investment strategy tends to be “slow investment return”. P2 and P17 are mainly distributed on the side of the PC2 shaft, with high N_mass_. P2 also has low ash-free calorific value, construction cost, and high specific leaf area. P2 tends to be a “fast investment return” energy investment strategy. Other clones balance energy investment and storage.

### 3.2. Response Characteristics of Leaf Indexes of Different Clones of Chinese Fir to Climate Change

The ash-free calorific value of most trees and shrubs was negatively related to the annual average temperature and precipitation. This is because plants need to maintain more energy in their organs to meet their heat needs and maintain their growth in unsuitable environments such as relatively low temperatures or insufficient precipitation [30]. The leaf calorific value of all nine tested Chinese fir clones was non-significantly correlated with the average monthly temperature but positively correlated with the average monthly precipitation. This indicates that when precipitation decreases, the calorific value content of Chinese fir leaves decreases, and energy metabolism accelerates to acquire resources quickly.

RDA results show a significant correlation between monthly average temperature, precipitation, and construction cost, indicating that when temperature increases, leaf construction costs increase to enhance the plant’s stress resistance, and the investment in growth increases to maintain self-growth [31]. Leaf N content is more sensitive to climate change [32]. In this study, the N content significantly correlates with the monthly average temperature, indicating that the leaves may improve their light and capacity to adapt to atmospheric warming by increasing energy investments, such as synthesizing photosynthetic enzymes. To a certain extent, the specific leaf area reflects the light capture area of the leaves and the expected return on resource acquisition. It is closely related to plants’ survival and growth strategies and reflects plants’ adaptive characteristics in different environments [33]. Large and thin leaves are more beneficial for plants to capture light and improve their photosynthetic rate. In contrast, plants with lower specific leaf area characteristics can better adapt to relatively harsh environments, resulting in increased investment in structural materials (such as thickening of cell walls), small and thick leaves, and reduced photosynthetic rate [34].

A significant positive relationship exists between monthly average temperature and specific leaf area, indicating that Chinese fir can increase its photosynthetic rate by increasing leaf area and reducing structural material investment to adapt to atmospheric warming. The above discussion shows that under the circumstances of temperature rise and precipitation decrease in 2020, Chinese fir leaves exhibit a tradeoff between energy investment in high- and low-energy components. Under high temperatures and little rain, the investment in energy used for leaf construction increases to enhance its stress resistance. At the same time, to ensure the average growth of Chinese fir seedlings, the speed of energy metabolism is accelerated, the N content and specific leaf area of the leaves increase, and the photosynthetic rate increases.

## 4. Materials and Methods

### 4.1. Overview of the Study Area

**Figure 6:**
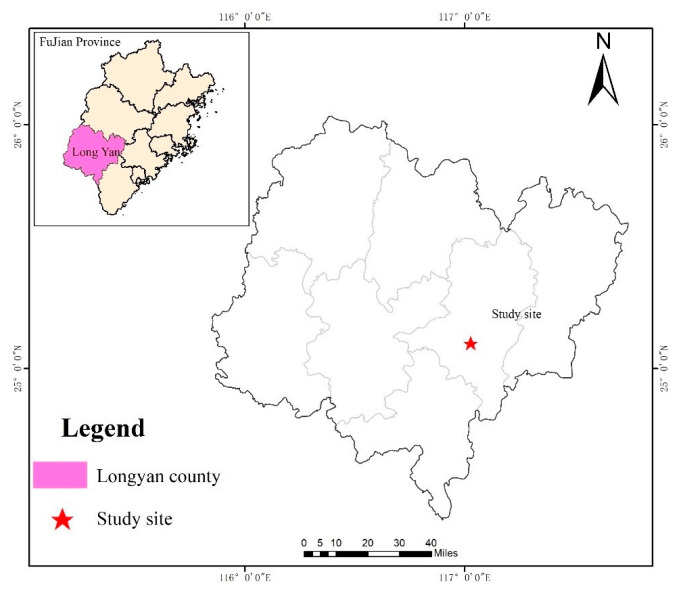
Location map of the study area.

The study area is in Baisha National Forest Farm, Shanghang, Longyan City, Fujian Province (25°01′ N, 116°20′ E) (Figure 6). It has a subtropical monsoon climate with distinct dry and wet seasons. The average annual temperature is 19.9 °C, the average annual precipitation days are 160 days, and the frost-free period is 285–301 days. The soil is mainly mountain red soil developed from coarse granite, the pH value was 3.68 on average, and the soil bulk density was 1.23 g/cm^3^. The contents of total phosphorus (TP), total potassium (TK), and total carbon (TC) in the soil were 0.17, 2.02, and 9.35 g/kg, respectively. The available phosphorus (AP) and potassium (AK) were respectively 3.31 and 34.09 mg/kg in soil depth of 0–40 cm [35].

In March 2019, one-year-old healthy and pest-free seedlings (nine Chinese fir clones) with consistent growth were selected for afforestation. Each clone was planted along the hillside in a longitudinal arrangement, with a spacing of 1.8 m × 2.0 m, open hole with 60 cm depth × 40 cm width × 40 cm length in size. After afforestation, it was thoroughly weeded and nurtured once to twice a year.

**Figure 7:**
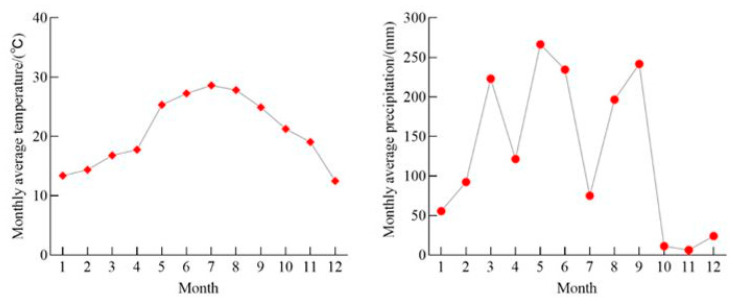
Change in monthly average temperature and precipitation in the study area in 2020.

According to the records of Shanghang Meteorological Observation Station (25°01′ N, 116°15′ E) nearest to the study area, the average annual temperature and precipitation data during the years 2010–2020 is shown in Table 4. To study the correlation between the characteristics of different Chinese fir leaves and climate factors after afforestation, the monthly temperature and precipitation in 2020 are shown in Figure 7. The temperature rose rapidly from May onward, and the average temperature in July reached a maximum of 28.61 °C. Since then, the temperature has decreased continuously, reaching a minimum of 12.49 °C in December. The monthly average precipitation reached the maximum of 266.42 mm in May, and the monthly average precipitation in June, August, and September also remained high. According to the local climate characteristics in the previous ten years, it could be seen that 2020 would be a year of high temperatures and little rain. Meteorological data were collected from China Meteorological Data Network (http://data.cma.cn/, accessed on 10 April 2023).

### 4.2. Test Materials

Nine tested Chinese fir clones, including L27, S4, S18, S22, P2, P17, P302, Yang020, and Yang061, were taken from the Chinese fir engineering technology research center of the State Forestry and Grassland Administration and Yangkou State–owned Forest Farm in Fujian Province. In October 2020, each tree of nine Chinese fir clones was measured to calculate the average tree height and ground diameter. The standard trees were selected on the basis of the calculation results. Three healthy, disease-free, standard trees were selected as the sampling objects for each clonal test forest. Then 30 g of the current year’s leaves from the top of the sunny side of the plant were collected as the test material and taken back to the laboratory for determination.

### 4.3. Determination of Specific Leaf Area, Ash Content, and Calorific Value

An EPSON V39 scanner was used to scan fresh leaves, and Image-J processing software was used to calculate leaf area. After recording the data, the leaves were put into an oven at 65 °C for drying. Once it reached a constant weight, the ratio of dry matter weight to leaf area was the specific leaf area (SLA). Ash content was determined by the muffle furnace dry ashing method. After grinding the dried sample, 1 g was accurately weighed, and this was ashed for 7 h in a muffle furnace at 700 °C. The ash mass/sample mass was the ash content (AC) (%).

Calorific value was divided into dry mass calorific value (GCV) and ash-free caloric value (AFCV). The ash-free caloric value was the calorific value obtained after the ash content removal. In this study, AFCV could eliminate the calorific value difference caused by different ash content and more accurately reflect the plant calorific value [19]. An oxygen cartridge (ZDHW-8000A) was used to measure the calorific value. Each sample was repeated three times, and the error was controlled within ±0.200 kJ/g. Before each determination, we calibrated with benzoic acid and controlled the ambient temperature at 25 °C.

(1) $$\mathrm{AFCV}=\frac{\mathrm{GCV}}{1-\mathrm{AC}}$$

### 4.4. Determination of Carbon (C) and Nitrogen (N) Content

The content of total C (C_mass_) and total N (N_mass_) was determined by the VARIO MAX CN C and N element analyzer produced by the German Elemental Company.

### 4.5. Calculation of Cost of Leaf Construction

The cost of leaf construction was calculated following the method of Williams [36].

(2) $${\mathrm{CC}}_{m}=\frac{\left(0.06968\mathrm{AFCV}-0.065\right)\left(1-\mathrm{AC}\right)+7.5\left(\frac{kN_{\mathrm{mass}}}{14.0067}\right)}{\mathrm{EG}}$$

CC_m_ is the construction cost, AFCV is the ash-free calorific value, AC is the ash content, EG is the growth efficiency taken as 0.87 [10], k is the redox state of N, the nitrate state was 5, and the ammonia state was −3. The average of the two oxidation forms was taken as the calculation result in this study.

### 4.6. Data Analysis

We used SPSS software for one-way ANOVA and Pearson correlation analysis of various indicators. Principal component analysis (PCA) was used to comprehensively analyze each index and explore the energy investment strategies of different clones. The association between C_mass_, N_mass_, AC, SLA, AFCV, CC_m_, and environmental factors (temperature, precipitation) was analyzed by redundancy analysis (RDA) using Canoco 5.0 software.

## 5. Conclusions

It is concluded that Chinese fir trees of all the tested clones performed with certain advantages in light storage and transformation. The calorific value and construction cost of the Chinese fir leaves were significantly correlated with the C, N, and ash content. P2 tends to adopt a “rapid investment return” strategy to obtain more resources among the nine tested Chinese fir clones. In contrast, L27 tends to adopt a “slow investment return” strategy with strong environmental adaptability. When the climate continues to be warm, clones L27 with strong adaptability to environmental stress can be selected for the planting of Chinese fir plantations to ensure the sustainable development of Chinese fir plantations. Climate change can significantly affect the growth of Chinese fir leaves. In high temperatures and little rain, Chinese fir leaves adjust their energy investment to adapt to climate changes.

However, plant adaptation to the environment is not only related to energy investment in leaves but also affected by their structural characteristics and physiological and biochemical activities. In addition, seasons may interact with climate change, affecting the growth of Chinese fir trees and energy investment in their leaves. In the future, it is necessary to comprehensively consider the impact of other factors (structural and physiological factors, biochemical characteristics, and seasonal variation) on leaf energy investment and further reveal the response mechanism of Chinese fir to climate change. Moreover, in future studies, the impact of climate change on the growth strategy and environmental adaptability of Chinese fir clones can be further discussed by measuring the photosynthetic data of the leaves.

## Figures and Tables

**Table 1:** Comparison of carbon content (C_mass_) of leaves among Chinese fir clones. Different lowercase letters indicate significant differences among clones (*p* < 0.05).

Clones	L27	S4	S18	S22	P2	P17	P302	Yang020	Yang061
**Carbon content (%)**	46.46 ± 0.34a	44.9 ± 0.67b	45.43 ± 0.20b	45.0 ± 0.53b	45.5 ± 0.12ab	45.6 ± 0.43ab	45.73 ± 0.93ab	45.39 ± 0.46b	45.64 ± 0.44ab

**Table 2:** Load of each index on two principal component axes. AC is ash content, AFCV is ash-free calorific value, C_mass_ is carbon content, N_mass_ is nitrogen content, SLA is specific leaf area, and CCm is leaf construction cost.

Index	AFCV	CC_m_	AC	C_mass_	N_mass_	SLA	Contribution Rate
**PC1**	−0.821	−0.772	0.719	−0.816	−0.054	−0.235	41.88%
**PC2**	0.233	−0.465	−0.602	−0.202	−0.981	−0.202	27.95%

**Table 3:** Scores of different clones on two principal component axes.

Clones	L27	S4	S18	S22	P2	P17	P302	Yang020	Yang061
**PC1**	−2.629	1.807	0.343	1.772	1.252	−0.979	−0.352	−0.780	−0.433
**PC2**	−0.224	1.403	0.981	−0.982	−1.529	−1.636	−0.822	1.290	1.529

**Table 4:** The annual average temperature and precipitation changes in the study area from 2010 to 2020.

Year	Annual Average Temperature (°C)	Annual Average Precipitation (mm)
2010	19.85	1786.31
2011	19.55	1400.52
2012	19.83	1980.95
2013	20.03	1825.30
2014	20.26	1424.96
2015	20.41	1781.80
2016	20.40	2473.20
2017	20.62	1563.26
2018	20.47	1392.85
2019	20.77	1779.63

## Data Availability

Data are available through the corresponding author upon reasonable request for academic purposes.
